# *Pseudomonas avellanae* : From Specific Pathogens to a Species Complex

**DOI:** 10.1111/mpp.70357

**Published:** 2026-10-19

**Authors:** Marco Scortichini, Tatjana Popović Milovanović, Renata Iličić

**Affiliations:** ^1^ CREA, Research Centre for Olive, Fruit and Citrus Crops Rome Italy; ^2^ Institute for Plant Protection and Environment Belgrade Serbia; ^3^ Faculty of Agriculture University of Novi Sad Novi Sad Serbia

**Keywords:** endophytic phase, European hazelnut, kiwifruit, *Pseudomonas* species complex, sour cherry, sweet cherry, tea

## Abstract

**Taxonomy:**

Bacteria; *Proteobacteria*, gamma subdivision; Order *Pseudomonadales*; Family *Pseudomonadaceae*; Genus *Pseudomonas*; *Pseudomonas avellanae* species complex, which includes different pathogenic lineages previously classified within the *P. syringae* complex, namely the pathovars *avellanae*, *theae*, *actinidiae*, and *morsprunorum* race 2.

**Microbiological Properties:**

As a whole, the *P. avellanae* species complex genome comprises 19.4% conserved genes and 80.6% flexible genome. Mobile genetic elements are present within the complex. Strains associated with bacterial canker of European hazelnut possess two plasmids, whereas multiple plasmids and megaplasmids can occur in the lineages associated with bacterial canker of kiwifruit and cherry. Fourteen type III secretion system effectors are shared by all lineages of the species, and each lineage apparently possesses specific effector proteins. Phytotoxins, such as phaseolotoxin, coronatine, and tabtoxin, can be produced by some lineages of *P. avellanae* . Siderophores are present in the *P. avellanae* species complex.

**Host Range:**

*Corylus avellana* , *Camellia sinensis* var. *sinensis* , *Actinidia chinensis* var. *chinensis*, *A. chinensis* var. *deliciosa*, *Prunus avium* , *P. cerasus* , *P. domestica* . A strain of *P. avellanae* has also been isolated from river epilithon.

**Disease Symptoms:**

*Pseudomonas avellanae* can cause severe symptoms, including bud necrosis (kiwifruit), leaf necrosis and wilting (hazelnut, tea, kiwifruit, cherry/plum), shoot and twig blight (hazelnut, tea, kiwifruit, cherry/plum), flower necrosis (kiwifruit), fruit necrosis (cherry/plum), trunk canker (hazelnut, kiwifruit, cherry/plum), and plant death (hazelnut, tea, kiwifruit).

**Epidemiology:**

Some *P. avellanae* lineages are favoured by air temperatures below 0°C. Penetration mainly occurs through stomata, leaf scars, and wounds induced by frost events. *P. avellanae* species complex exhibits both epiphytic and endophytic phases. During summer, very high temperatures can reduce the multiplication and dispersal of the pathogen. The global circulation of latently infected propagative plant material largely favours the spread of *P. avellanae* lineages across different cultivation areas.

**Detection:**

Pathogens belonging to this complex can be detected in both symptomatic and asymptomatic plant tissues (e.g., twigs, buds, and cankers) using various conventional techniques and molecular tools (standard‐, duplex‐, TaqMan‐, real‐time PCR, LAMP, MLSA). Depending on the targeted symptoms, pathogenicity tests may be conducted on leaf, leaf scars or woody tissues.

**Disease Control:**

Disease management is based on spray treatments to the crown using copper compounds applied in spring and autumn, as well as after harvest, during leaf fall, and following hail, frost events, and pruning. Balanced plant nutrition is important to reduce the risk of severe infection. Alternative promising strategies include the application of biocontrol agents, natural products, and bacteriophages.

## Graphical abstract

The Pseudomonas avellanae species complex includes pathogens formerly classified within Pseudomonas syringae, namely the pathovars avellanae, actinidiae, theae, and morsprunorum race 2.

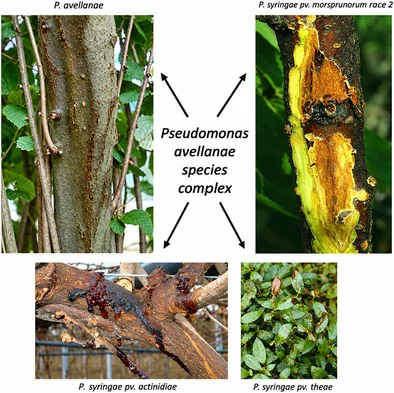

## 1 Introduction

In 1915, a severe disease of tea plants ( *Camellia sinensis* var. *sinensis* ) was reported in Japan, and *Bacillus theae* was indicated as the causal agent (Hori 1915). In 1955, the disease was named bacterial shoot blight of tea, with *Pseudomonas theae* proposed as the causal agent (Okabe and Goto 1955). In 1978, following the introduction of the pathovar concept, *P. syringae* pv. *theae* was used to refer to this disease (Young et al. 1978). In 1975, a variant of *P. morsprunorum* affecting sweet cherry was described in England (Freigoun and Crosse 1975). In 1976, extensive twig and branch dieback, accompanied by longitudinal cankers along the trunk, was observed in European hazelnut ( *Corylus avellana* ) orchards in northern Greece. The disease was attributed to *P. syringae* *sensu lato* (Psallidas and Panagopoulos 1979), and in 1993, *P. syringae* pv. *avellanae* was formally described as the causal agent of bacterial canker of European hazelnut (Psallidas 1993). In the same period, a very similar disease was observed in several European hazelnut orchards in central Italy (Viterbo province), where *P. syringae* pv. *avellanae* was isolated and characterised in 1994 (Scortichini and Tropiano 1994). In 1980, a severe disease of green‐fleshed kiwifruit orchards ( *Actinidia chinensis* var. *deliciosa*) was observed in Japan; in 1989, the bacterial canker of kiwifruit was formally described, and *P. syringae* pv. *actinidiae* was indicated as the causal agent (Takikawa et al. 1989).

In 1996, *P. syringae* pv. *avellanae* was formally elevated to species level as *P. avellanae* (Janse et al. 1996). Furthermore, based on DNA–DNA relatedness among strains belonging to the *P. syringae* complex, the seminal paper of Gardan et al. (1999) placed *P. avellanae* and *P. syringae* pv. *theae* within genomospecies 8, with *P. avellanae* as the reference species. Strains of *P. syringae* pv. *actinidiae* were not included in this study. The relevant taxonomic similarity of *P. syringae* pv. *actinidiae* strains with genomospecies 8 was pointed out in subsequent studies (Scortichini et al. 2002; Manceau and Brin 2003). Extensive genomic analyses have confirmed the close taxonomic relationships among the causal agents of bacterial shoot blight of tea, bacterial canker of European hazelnut, and bacterial canker of kiwifruit (Scortichini et al. 2013; Marcelletti and Scortichini 2014; Gomila et al. 2017). Interestingly, this species also includes *P. syringae* pv. *morsprunorum* race 2 (Scortichini et al. 2013; Marcelletti and Scortichini 2014, 2019; Nowell et al. 2016; Gomila et al. 2017), one of the causal agents of bacterial canker of sweet and sour cherry, and European plum ( *Prunus avium* , *P. cerasus* , *P. domestica* ) (Freigoun and Crosse 1975; Hulin, Jackson, et al. 2020). This pathovar belongs to phylogroup PG01 of the *P. syringae* complex, according to Berge et al. (2014). According to these studies, four distinct *P. syringae* pathovars can now be included in a taxonomically delineated species complex, defined as “a single species composed of distinct populations capable of infecting a limited number of host plants” (Stolp et al. 1965; Doudoroff and Palleroni 1974).

The distinct genomic lineages (i.e., populations *sensu* Stolp et al.) of the *P. avellanae* species complex cause severe economic losses in different host plants across various geographic areas. Significant examples include more than 1000 ha of hazelnut orchards initially destroyed by bacterial canker of European hazelnut in central Italy (Scortichini 2002). Economic losses exceeded €2 million in Italy in 2009 at the onset of the epidemic and later reached more than €400 million in New Zealand in 2014 due to bacterial canker of kiwifruit (Narouei‐Khandan et al. 2022). Notably, this disease is considered pandemic, as it has spread to all major kiwifruit‐growing regions worldwide (Nunes da Silva et al. 2022). Bacterial canker of cherry also accounts for substantial economic losses, with reports of up to 75% yield loss in Oregon, United States (Spotts et al. 2010). Interestingly, a strain isolated in the United States from river epilithon (i.e., rock‐attached biofilms composed of algae, diatoms, rotifers, bacteria, and nematodes) also belongs to *P. avellanae* (Morris et al. 2007; Turco et al. 2022).

This review concisely addresses the taxonomy, biology, host range, disease symptoms, epidemiology, detection, and disease control of the *P. avellanae* species complex. In this review, we use the term “ *Pseudomonas avellanae* species complex” to describe the genetically related, biologically and genomically differentiated lineages assigned to the genomic species *P. avellanae* . Such lineages belong to the formally recognised former pathovars: *P. syringae* pv. *avellanae*, *P. syringae* pv. *theae*, *P. syringae* pv. *actinidiae* and *P. syringae* pv. *morsprunorum* race 2. This should also facilitate reading and comparison with the published literature.

## 2 Taxonomy

*Pseudomonas avellanae* as a distinct species appears quite robust. DNA–DNA relatedness, average nucleotide identity (ANI) coupled with TETRA analysis, genome‐to‐genome distance calculator (GGDC), 16S rRNA gene sequencing, multilocus sequence type analysis (MLSA), as well as whole‐genome sequencing (WGS) and comparative analyses all provide clear evidence that this species can be distinguished from the *P. syringae* complex (Janse et al. 1996; Gardan et al. 1999; Scortichini et al. 2002; Scortichini et al. 2013; Gomila et al. 2017). The inclusion of three *P. syringae* pathovars, namely *theae*, *actinidiae* and *morsprunorum* race 2, within the *P. avellanae* cluster is also taxonomically coherent. Phylogenetic analyses based on the concatenation of more than 2000 orthologous genes, as well as the MLSA, support this inclusion (Nowell et al. 2016; Gomila et al. 2017; Hulin, Mansfield, et al. 2018). In addition, ANI and TETRA analyses indicated similarity values among strains of the *P. avellanae* cluster, including the three pathovars, ranging from 97.59% to 99.92%, with *P. syringae* pv. *tomato* being the most closely related pathogen (Scortichini et al. 2013). A split consensus network based on concatenated nucleotide sequences of seven housekeeping genes also indicated a close relationship between *P. syringae* pv. *tomato* strains and *P. avellanae* (Marcelletti and Scortichini 2014). Similarity between this pathovar and *P. avellanae* was also revealed by DNA–DNA analysis (Gardan et al. 1999). The same overall results were obtained by Gomila et al. (2017), who, using a phylogenomic approach based on ANI and UPGMA analyses, clearly demonstrated the distinctiveness of the *P. avellanae* cluster, which also includes the three *P. syringae* pathovars mentioned above (Figure 1). These results were also obtained from the comparison of concatenated amino acid sequences of 219 monocopy proteins from the core genome of 127 genomes of the *P. syringae* complex, which clearly showed the separation of the *P. avellanae* species complex from all the other phytopathogenic pseudomonads (Gomila et al. 2017). These analyses also indicate *P. syringae* pv. *tomato* as the most closely related pathogen. Additional analyses further confirmed the robustness of this species complex, showing a minimal intracluster MLSA similarity of 99.0% and a minimal intracluster core genome similarity of 99.2% (Gomila et al. 2017). The strain isolated from the river epilithon also belongs to the *P. avellanae* species complex and is closely related, but not identical, to strains obtained from hazelnut based on ANI analysis (Turco et al. 2022). The strains of the *P. avellanae* species complex can also be phenotypically circumscribed using MALDI‐TOF mass spectrometry (Mulet et al. 2012).

**FIGURE 1:**
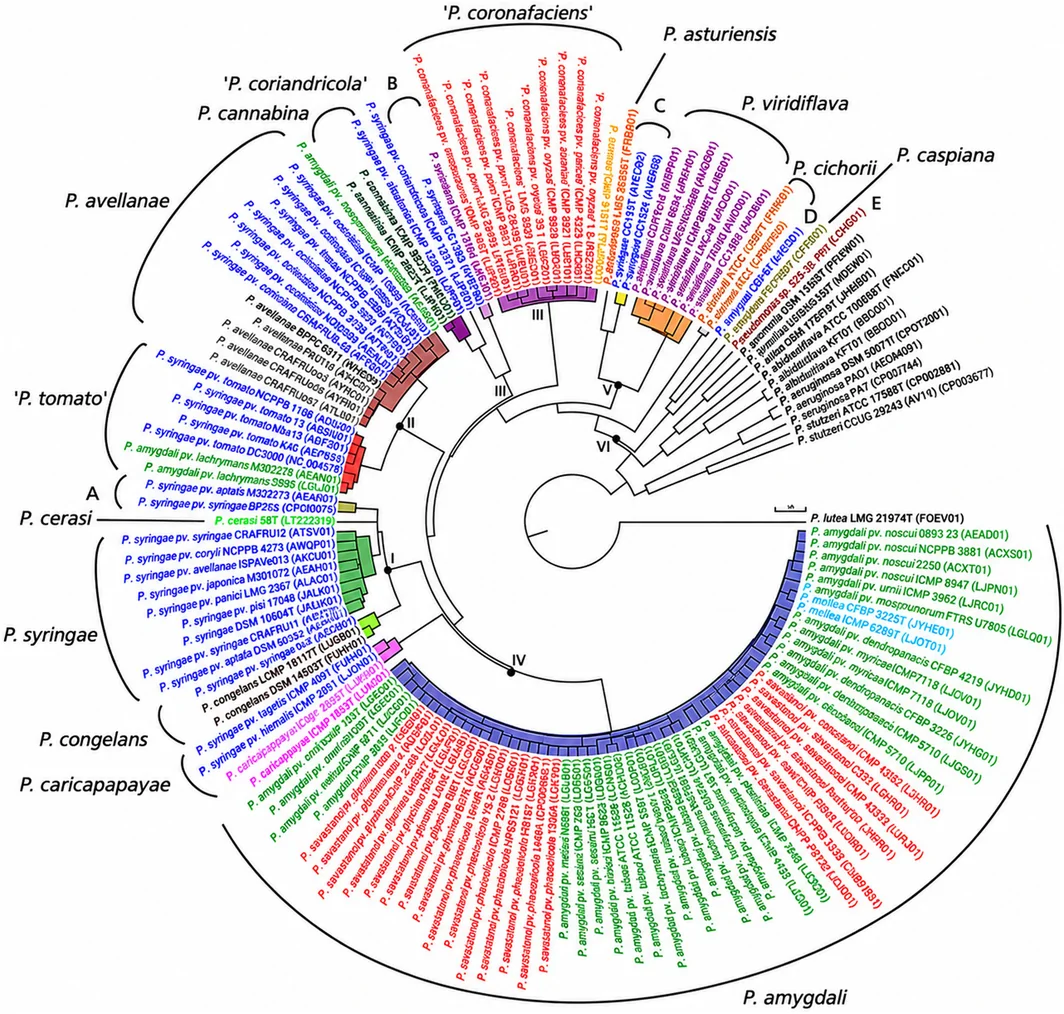
Dendrogram based on ANI values and UPGMA analysis of 139 genomes of pseudomonads belonging to the *Pseudomonas syringae* species complex. The *Pseudomonas avellanae* species complex includes the *P. syringae* pathovars *theae*, *actinidiae*, and *morsprunorum* race 2, with '*Pseudomonas tomato'* as the most closely related species (from Gomila et al. 2017).

## 3 Microbiological Properties

*Pseudomonas avellanae* appears to be widely distributed worldwide, as it has been isolated from different sources, including natural ones (i.e., river epilithon) and wild plants of the same species as cultivated hosts, in Asia, Europe and North America. As a whole, the *P. avellanae* species complex genome comprises 19.4% conserved genes and 80.6% flexible genome. Notably, the proportion of the flexible genome in this species is among the highest within the *P. syringae* complex, together with *P. amygdali* (i.e., 82.3%) (Gomila et al. 2017). Within this species complex, the different lineages (i.e., the four former *P. syringae* pathovars *avellanae*, *theae*, *actinidiae* and *morsprunorum* race 2) exhibit distinct microbiological characteristics. An interesting feature distinguishing the different *P. avellanae* lineages is the occurrence of the WHOP (woody host *Pseudomonas syringae* ) genomic region, which is involved in virulence and is present in lineages causing bacterial canker of kiwifruit and cherry, but absent in the lineage causing bacterial canker of European hazelnut (Turco et al. 2022).

Strains isolated from symptomatic European hazelnut in Greece and Italy can be differentiated according to their geographical origin using molecular typing methods such as repetitive sequence PCR and MLSA (Scortichini et al. 1998; Popović Milovanović et al. 2026). Within the former *P. syringae* pv. *actinidiae*, five different biovars are recognised (1, 2, 3, 5 and 6), which are differentiated based on virulence, single‐nucleotide polymorphisms, the occurrence of phytotoxins, or specific type III secretion system (T3SS) effector proteins (Scortichini et al. 2023). The former *P. syringae* pv. *morsprunorum* race 2 is traditionally differentiated from race 1 and from *P. syringae* pv. *syringae* based on specific biochemical tests such as gelatin and aesculin hydrolysis, tyrosinase activity, utilisation of tartaric and lactic acid, as well as specific pathogenicity assays (i.e., lesions on *Syringa vulgaris* leaves, colonisation of sweet cherry leaf scars, necrosis on sour cherry fruits, necrotic lesions on sour cherry and *P. domestica* leaves, and lesions on lemon fruits) and the production of syringomycin (Bultreys and Kaluzna 2010).

A basic effector repertoire of the T3SS of the *P. avellanae* species complex is shown in Figure 2. Fourteen effectors are shared by all lineages of the species, and each lineage apparently possesses a specific effector protein. Further analyses including additional strains identified specific T3SS effectors in lineages infecting European hazelnut (i.e., HopAA1, Hop AH1, HopAI1, HopB2 and HopM) (Turco et al. 2022). The T3SS effector repertoire has been studied in detail in the lineage corresponding to *P. syringae* pv. *actinidiae*. A list of T3SS effectors present in virulent and less virulent strains associated with bacterial canker of kiwifruit is shown in Table 1 (Choi et al. 2017). This lineage is capable of modulating and coordinating gene expression during the early phases of host colonisation. Nutrient acquisition (mainly phosphate) in the first 3 h following contact with the host plant is mediated by the type VI secretion system (T4SS). Subsequently, within 24 h, T3SS effectors are involved in subverting plant defence mechanisms and initiating infection. This is followed by alginate production and increased nutrient acquisition (McAtee et al. 2018). Upon colonisation of the apoplast, the host plant rapidly activates the production of peroxidase, β‐galactosidase, and heat shock proteins, followed by chitinases and pathogenesis‐related proteins (Petriccione et al. 2014). A substantial number of T3SS effectors (i.e., 24–34) is also present in the lineage *morsprunorum* race 2, located either on the chromosome or on plasmids (Hulin, Armitage, et al. 2018).

**FIGURE 2:**
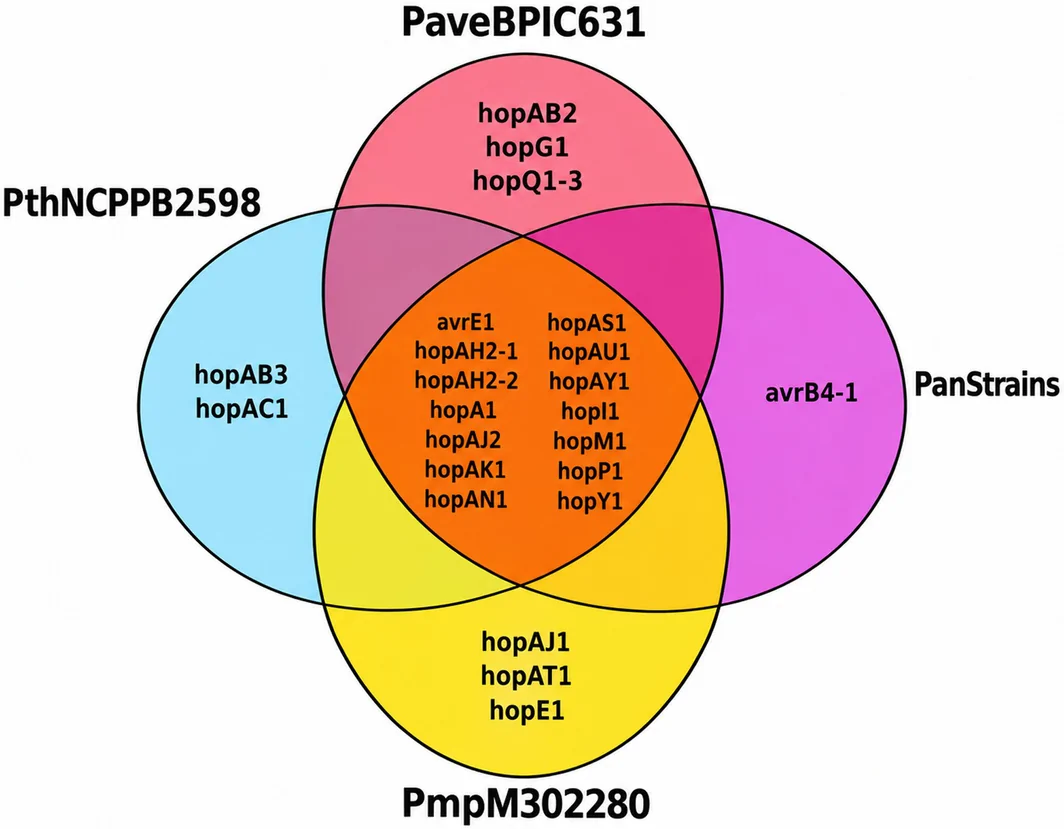
Venn diagram of the basic type III secretion system (T3SS) effector gene complements of the *Pseudomonas avellanae* species complex. The diagram is based on a comparison with the corresponding complements of other sequenced plant‐pathogenic pseudomonads and shows complete identity in reciprocal comparisons. Pave: *Pseudomonas avellanae* ; Pth: *Pseudomonas syringae* pv. *theae*; Pan: *P. syringae* pv. *actinidiae*; Pmp: *P. syringae* pv. *morsprunorum* race 2 (adapted from Scortichini et al. 2013).

**TABLE 1:** Repertoire of type III secretion system (T3SS) effectors in virulent (V13) and less virulent (LV5) strains of *Pseudomonas syringae* pv. *actinidiae* (adapted from Choi et al. 2017).

Effectors	*Psa*‐V13	*Psa*‐LV5	Amino acid identity (%)	Predicted protein size (kDa)
AvrB4‐1	+		NA	36.0
AvrD1	+			34.6
AvrE1	+	+	97.1	195.3
AvrPto5	+			17.2
AvrRpm1	+			24.9
HopA1	+^a^	+		22 (V13)/42.2 (LV5)
HopD1	+			34.6
HopE1		+		24.1
HopF1		+		21.9
HopF4b (HopF2)	+			22.0
HopH1	+			24.3
HopI1	+			49.3
HopM1	+			75.9
HopN1	+	+	99.6	38.7
HopO1		+		32.6
HopQ1	+			48.8
HopR1	+	+	99.0	210.3
HopS1		+		19.9
HopS2	+	+	94.9	18.7
HopT1		+		41.5
HopW1	+^a^	+		40.8 (V13)/82.8 (LV5)
HopX1		+		40.6
HopX2		+		38.0
HopX3	+			40.9
HopY1	+	+		30.9
HopZ3	+			45.1
HopAA1	+^a^	+		21.8 (V13)/50.4 (LV5)
HopAB3		+		62.6
HopAE1	+	+	99.8	126.6
HopAF1	+	+		23.4
HopAF1‐2		+		30.9
HopAG1		+		77.6
HopAH1	+	+	91.2	42.8
HopAI1^b^	+	+	81.6	29.7
HopAM1‐1	+			31.3
HopAO2	+			38.1
HopAR1		+		28.5
HopAS1	+	+	98.9	149.0
HopAU1	+			88.2
HopAV1	+			123.5
HopAW1	+			24.9
HopAY1^c^	+	+	91.9	27.1 (V13)/35.4 (LV5)
HopAZ1	+	+	99.5	24.7
HopBB1‐1 (2)	+			30.7
HopBN1	+			56.8

*Note:* “+” means present; “NA” means not analysed.

^a^ Effectors highly truncated in *Psa* V13 indicating a pseudogene.

^b^ Amino acid identity between alleles from *Psa* V13 and LV5 lower than 90%.

^c^ *Psa* V13 version has shorter amino acid sequence.

Mobile genetic elements are present in the *P. avellanae* species complex. Strains associated with bacterial canker of European hazelnut possess two plasmids (Turco et al. 2022), whereas multiple plasmids and megaplasmids can occur in lineages associated with bacterial canker of kiwifruit and cherry (McCann et al. 2017; Hulin, Armitage, et al. 2018). Mobile genetic elements and horizontal gene transfer play an important role in determining pathogenicity in the *P. avellanae* species complex.

An exchange of T3SS effectors located on plasmids has been reported between different pseudomonads affecting cherry trees, likely contributing to convergent pathogenicity towards the same host plant among taxonomically divergent *Pseudomonas* spp., such as *P. avellanae* and *P. amygdali* , the latter including the former named as *P. syringae* pv. *morsprunorum* race 1 (Hulin, Armitage, et al. 2018). Exchange of variable genomic regions also occurs between the closely related species *P. avellanae* and *P. syringae* pv. *tomato* (Figure 3). Different integrative conjugative elements (ICEs) of genomic islands, identified in strains of the *P. syringae* species complex, largely contribute to differentiating *P. syringae* pv. *actinidiae* populations from different geographical areas, all belonging to biovar 3 (Butler et al. 2013). Some phytotoxins are also present within the species complex, but their occurrence varies among lineages. The presence or absence of phytotoxins is a key factor in discriminating biovars of strains associated with bacterial canker of kiwifruit. In particular, phaseolotoxin and coronatine may be present or absent, being encoded in the chromosome or in a megaplasmid, respectively (Fujikawa and Sawada 2019). Tabtoxin occurs in strains associated with bacterial canker of hazelnut, cherry and kiwifruit, whereas coronatine, phaseolotoxin, syringomycin and syringopeptin are absent in these lineages (Marcelletti and Scortichini 2015; Turco et al. 2022). Siderophores are present in the *P. avellanae* species complex. Bacterioferritin, ferritin, pyoverdine, pyridine and yersiniabactin occur in strains associated with bacterial canker of hazelnut (Marcelletti and Scortichini 2015), whereas pyoverdine, enterobactin and yersiniabactin occur in strains associated with bacterial canker of kiwifruit (Marcelletti et al. 2011). The ice nucleation gene cluster is only partially present in the *P. avellanae* species complex (Turco et al. 2022).

**FIGURE 3:**
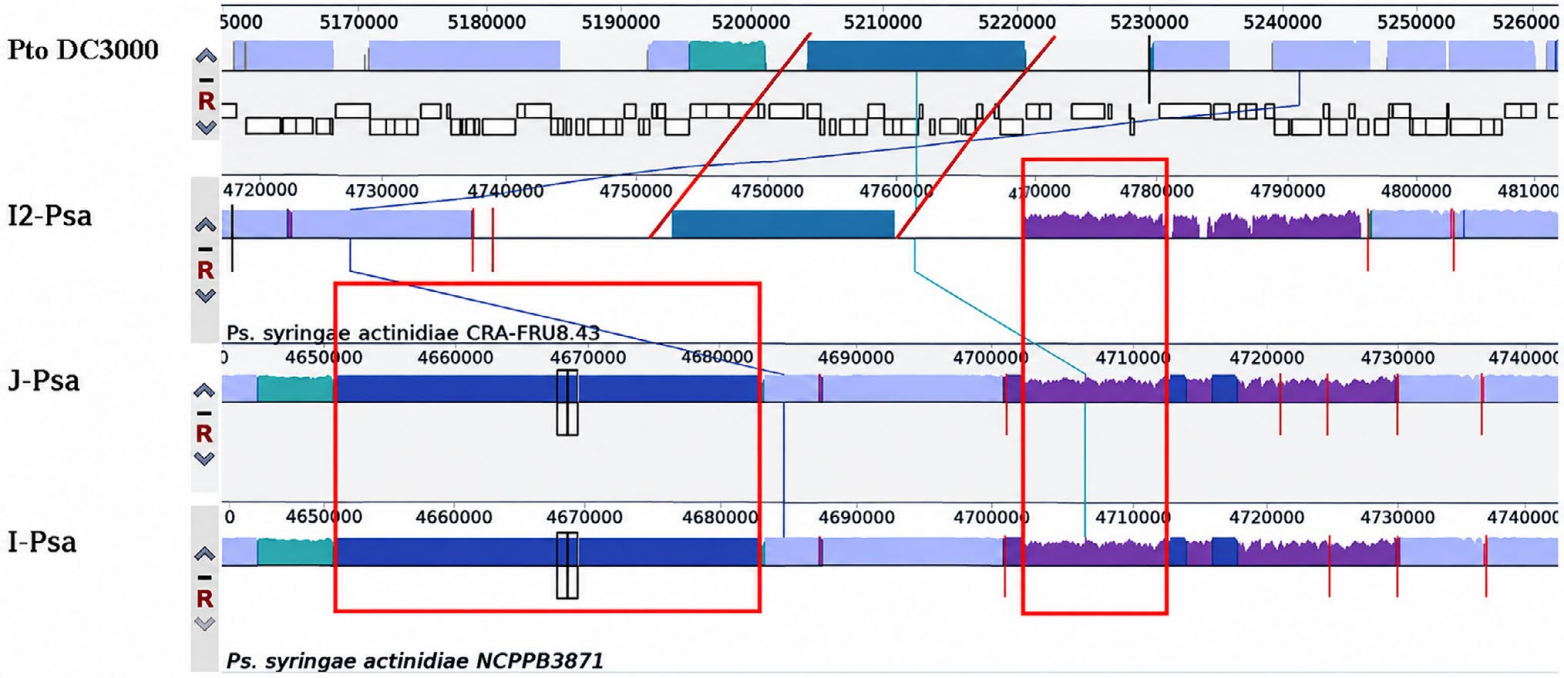
Partial genome alignment between strains associated with bacterial canker of kiwifruit (i.e., *Pseudomonas syringae* pv. *actinidiae*) (I‐Psa: Isolated in Italy in 1992; I2‐Psa: Isolated in Italy in 2008; J‐Psa: Isolated in Japan in 1984) and *P. syringae* pv. *tomato* DC3000. The violet segments (right) indicate variable region 3, present in all three *P. syringae* pv. *actinidiae* strains but absent in *P. syringae* pv. *tomato* DC3000, whereas the dark blue segments (left) correspond to variable region 2, present in J‐Psa and I‐Psa but absent in I2‐Psa and *P. syringae* pv. *tomato* DC3000. The figure also shows several shorter regions (i.e., light green segments), which likely represent examples of horizontal gene transfer as well as a region in common between *P. syringae* pv. *tomato* DC3000 and I2‐Psa (blue green segments) (adapted from Marcelletti et al. 2011).

## 4 Host Range and Disease Symptoms

The host range of the *P. avellanae* species complex is shown in Table 2. The different lineages induce various disease symptoms in woody and shrub plant species such as *Corylus avellana* , *Camellia sinensis var. sinensis* , *Actinidia chinensis* var. *deliciosa*, *A. chinensis* var. *chinensis*, *Prunus avium* , *P. cerasus* , and *P. domestica* .

**TABLE 2:** Host range, symptoms induced by different lineages of the *Pseudomonas avellanae* species complex, and primers used for their detection. Lineage names refer to the former *P. syringae* pathovar names.

Species	Host	Symptom	PCR	Molecular identification		
				Primers	Sequence	References
*P. avellanae*	*Corylus avellana*	Leaf necrosis and wilting, shoot and twig blight, trunk canker, plant death	Standard	PAV 1 PAV 22	5′‐GGCGACGATCCGTAACTGGTCTGAGA‐3′ 5′‐TTCCCGAAGGCACTCCTCTATCTCTAAAG‐3′	Scortichini and Marchesi (2001)
			Standard	WA WC	5′‐TCCACAGGACGCCAGCAAGA‐3′ 5′‐TGCGGTGTTACGCCACCATC‐3′	Loreti and Gallelli (2002)
			TaqMan	Pavel‐F Pavel‐R Pavel‐MGB	5′‐GCACTTTAAGTTGGGAGGAA‐3′ 5′‐CAGAGTTAGCCGGTGCTTA‐3′ 5′‐AATACGTATCTGTTTTG‐3′‐MGB	Gervasi and Scortichini (2009)
			Real‐time	Pav‐hsdF Pav‐hsdR	5′‐GAGGCGCAGGGGCTGATGTT‐3′ 5′‐TGGAGGACATCTTGCTTCTTCT‐3′	Turco et al. (2023)
*P. syringae* pv. *theae*	*Camellia sinensis*	Leaf necrosis and wilting, shoot and twig blight, plant death				
*P. syringae* pv. *actinidiae*	*Actinidia chinensis* var. *chinensis* *Actinidia chinensis* var. *deliciosa*	Bud necrosis, leaf necrosis and wilting, shoot and twig blight, flower necrosis, trunk canker, plant death	Standard	PsaF1 PsaR2	5′‐TTTTGCTTTGCACACCCGATTTT‐3′ 5′‐CACGCACCCTTCAATCAGGATG‐3′	Rees‐George et al. (2010)
			Standard	PsaF3 PsaR4	5′‐ACCTGGTGAAGTTGGTCAGAGC‐3′ 5′‐CGCACCCTTCAATCAGGATG‐3′	
			Duplex	KN‐F KN‐R AvrPtoB‐LF AvrPtoB‐LR	5′‐CACGATACATGGGCTTATGC‐3′ 5′‐CTTTTCATCCACACACTCCG‐3′ 5′‐GGAGAGGATCAGCATATG‐3′ 5′‐TCAGGGGACTATTCTAAAAG‐3′	Gallelli et al. (2011)
			Simplex	P0F P6R	5′‐CTGCAACAGGCGACGGCGAGGC‐3′ 5′‐CATAGGCTTCTGGTTTTCTTCCTGATCC‐3′	Gallelli et al. (2014)
			Real‐time	P3F P5R1	5′‐GGTTTCGGACACCGCAGGTTCTACCGAG‐3′ 5′‐CTTCCTGATCCCCGTTACCCATCGAC‐3′	Gallelli et al. (2014)
			Real‐time	*hopA1* Psa_A1F2 Psa_A1 R1	5′‐GCCTCGATGTCGGCGC‐3′ 5′‐ATTCGATAGAAGAACTTCTTTGCGTTT‐3′	Andersen et al. (2018)
			Real‐time	hrpZ5 Psa_Z5 F2 Psa_Z5 R2	5′‐ACAACTTCAGGCTACAATACTTACGC‐3′ 5′‐CTCAGGATGCGTTTCGGTTAC‐3′	Andersen et al. (2018)
			LAMP	F3 B3 FIP BIP loopF loopR	5′‐GGCTCTCCTAGCAAGCATAC‐3′ 5′‐TGAGAAGGGACGCAACCA‐3′ 5′‐CGGATTCGCAACGCTCCAAGATCTGCTGA GCACGTTGGTC‐3′ 5′‐ACTCTTCCGCAACGAGTTTGGGTCCCCAC ATGGAGTTGTCT‐3′ 5′‐TGCCGATCGAGTATCCATTTCCT‐3′ 5′‐AGCCTTTTCCGAACGGTCT‐3′	Ruinelli et al. (2017)
			LAMP (biovar 3)	F3 B3 FIP BIP loopF loopR	5′‐GCTATGGAATCCATTGCGGT‐3′ 5′‐CGCATCTGCTGGATCATCC‐3′ 5′‐ATCCCTTGCCCAGCACGAACATGAGGTCG AGGTGTCTGA‐3′ 5′‐ATCCACAGTGGGTACACGGACGGGGGCA CCTTCTTTCTTGG‐3′ 5′‐GGCCTTTTCAATGCGGTCAATATCC‐3′ 5′‐GGTGTCAATCTGGTGGTGCAT‐3′	Ruinelli et al. (2017)
*P. syringae* pv. *morsprunorum* race 2	*Prunus avium* *Prunus cerasus* *Prunus domestica*	Leaf necrosis and wilting, shoot and twig blight, fruit necrosis, trunk canker	Standard	Psm2‐8F/ Psm2‐8R	5′‐CTTTTTAGATGGTGAGGTTTTGTA‐3′ 5′‐ACTTTCGGATCATCGTTTTCTA‐3′	Kałużna et al. (2016)
			Real‐time	Psm2‐1F‐RT/ Psm2‐1R‐RT	5′‐GGTTTGCCTTTTCCTCAG‐3′ 5′‐ATTGCATTACTTCTTTGTTGC‐3′	Kałużna et al. (2016)

The main symptoms are as follows: bud necrosis (kiwifruit), leaf necrosis and wilting (hazelnut, tea, kiwifruit, cherry/plum), shoot and twig blight (hazelnut, tea, kiwifruit, cherry/plum), flower necrosis (kiwifruit), fruit necrosis (cherry/plum), trunk canker (hazelnut, kiwifruit, cherry/plum) and plant death (hazelnut, tea, kiwifruit) (Table 2 and Figure 4). Notably, plants infected with bacterial canker of kiwifruit may exhibit bacterial exudates during early spring. For all the lineages of the *P. avellanae* species complex, the symptoms are more evident in spring, autumn and late winter, whereas summer is not conducive to either pathogen multiplication or disease progression. Under artificial inoculation, different lineages of the *P. avellanae* species complex exhibit differential pathogenicity towards selected host plant organs. Strains associated with hazelnut do not cause symptoms when inoculated onto cherry fruitlets and peach leaves. However, they can induce water‐soaked lesions on almond leaves. A strain associated with tea can induce water‐soaked lesions on peach and almond leaves. Strains associated with kiwifruit can induce water‐soaked lesions on cherry fruitlets, whereas strains from cherry can induce such lesions on cherry fruitlets, as well as on peach and almond leaves (Ruinelli et al. 2019). A strain resembling *P. avellanae* has been isolated from Cornelian cherry (*Cornus mas*) that caused leaf spotting (Kałużna 2019).

**FIGURE 4:**
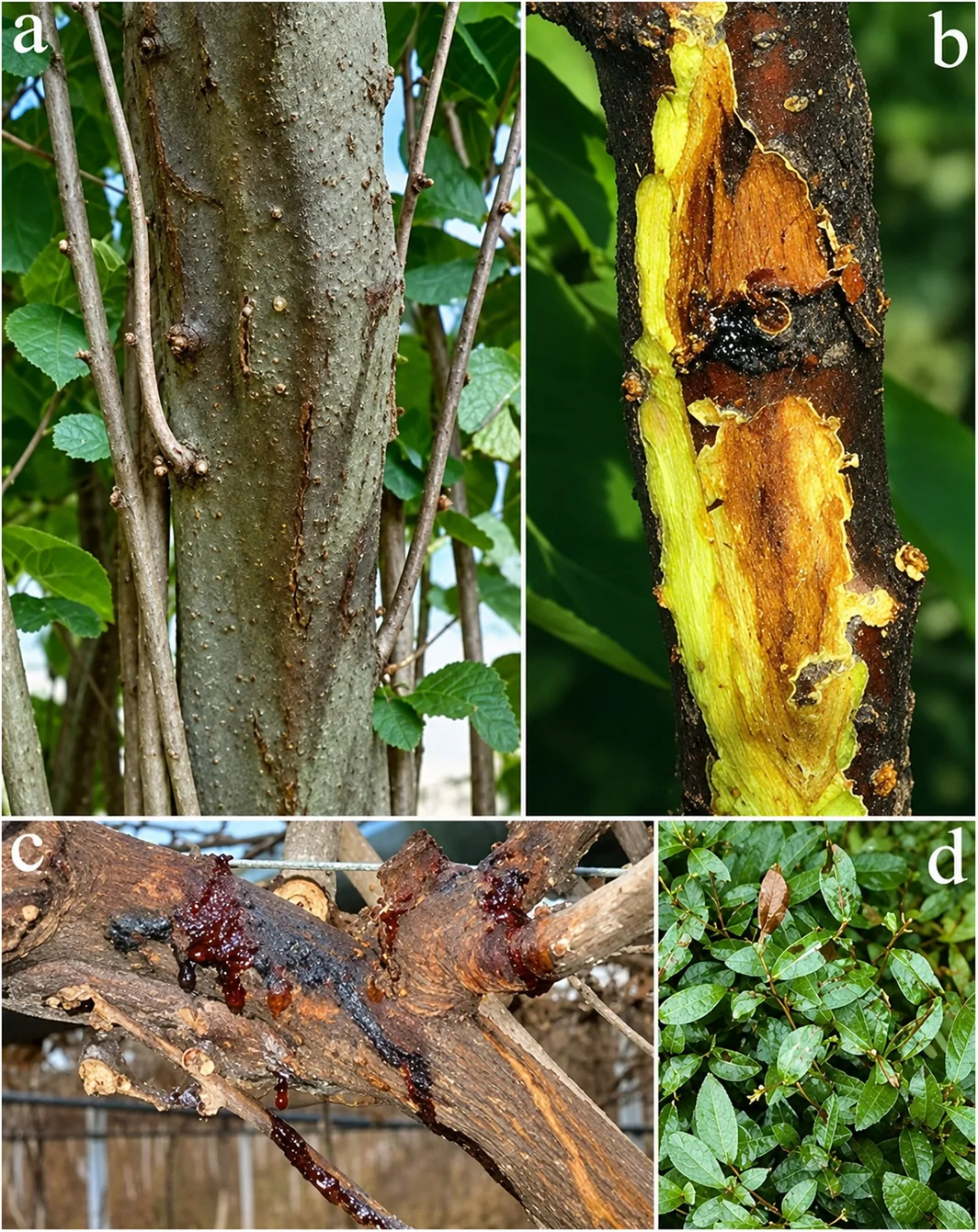
Representative disease symptoms caused by different lineages of *Pseudomonas avellanae* species complex in (a) European hazelnut, (b) sweet cherry, (c) yellow‐fleshed kiwifruit, and (d) tea. In sweet cherry, canker lesions on branches may also harbour strains of *Pseudomonas syringae* pv. *syringae* and *Pseudomonas amygdali* pv. *morsprunorum* race 1. Photo credit: (d) M. Goto, University of Shizuoka, Japan.

## 5 Epidemiology

Detailed information is available on the epidemiology of the *P. avellanae* species complex. Despite the absence of a complete gene cluster encoding ice nucleation activity, some *P. avellanae* lineages are favoured by air temperatures below 0°C. The pathogenicity of strains associated with kiwifruit and tea is strongly promoted by frost events (Tomihama et al. 2009; Ferrante and Scortichini 2014), whereas frost damage to plants strongly favours colonisation by the strains associated with hazelnut (Scortichini 2002). The lineage associated with cherry/plum is favoured by air temperatures around 15°C (Bultreys and Kaluzna 2010). The lineage associated with kiwifruit displays an epiphytic phase in spring on buds, leaves, twigs, and flowers. However, the buds colonised during the previous season may fail to develop leaves (Donati et al. 2020). On leaves, optimal conditions for growth are found at the base of glandular trichomes, as these structures retain water and provide nutrients such as sugars, proteins, and secondary metabolites. Leaf wetness is an essential prerequisite for further plant colonisation, which occurs through stomata and floral organs (i.e., stigma and anthers). Upon colonisation of the leaf apoplast, this lineage enters its endophytic phase and, through leaf veins and the petiole, reaches the twig. Visible symptoms (i.e., leaf and shoot blight) occur after the pathogen reaches cell densities ranging from 10^3^ to 10^4^ CFU g/fresh weight, with optimal air temperatures of 21°C–25°C (Donati et al. 2020). During summer, increased temperatures and reduced leaf wetness limit pathogen multiplication. In autumn, additional systemic invasion may occur through leaf and fruit abscission scars and pruning cuts, a process strongly favoured by rain and wind. This lineage can migrate within the plant both acropetally and basipetally and may also colonise the root system. It overwinters in infected plant organs, fallen leaves and pruning debris, and can also survive on leaves of wild or ornamental plants such as *Bellis perennis* , *Chenopodium album* , *Taraxacum officinale* , *Pinus radiata* , *Cryptomeria japonica* and *Chamaerops humilis* (Donati et al. 2020). At the end of dormancy, high root pressure may displace pathogen cells from the roots to the trunk and buds, leading to systemic infection of shoots. During this period, the pathogen can be released from infected plants through exudates containing bacterial cells, facilitating disease spread to neighbouring plants via wind. In spring, the pathogen may also colonise the anthers internally, leading to pollen infection, which presents another important pathway for environmental dissemination (Donati et al. 2020).

For strains associated with hazelnut, acidic soils, often combined with high aluminium content (i.e., approximately 20%), present important predisposing factors that increase pathogen virulence. In both the Viterbo area and northern Greece, the most severe symptoms (i.e., rapid decline and plant death) were observed in hazelnut orchards with soil pH below 5.0 (Scortichini 2002). Penetration through leaf scars, favoured by rain and wind, presents another key factor in pathogen dissemination in autumn. Pathogenicity assays confirmed this route of entry by showing a high success rate in reproducing field symptoms (Scortichini and Tropiano 1994; Popović Milovanović et al. 2026). As mentioned above, winter and spring frost, by causing wounds on trunks and branches, favours subsequent colonisation by bacterial cells carried by rain and wind. Once inside the tree, similarly to strains infecting kiwifruit, the pathogen can move acropetally and basipetally, reaching either the apical parts or the root system. Consequently, large‐scale disease spread occurs through the circulation of latently infected propagative material derived from apparently healthy mother plants (Scortichini and Tropiano 1994). This dissemination pathway was also confirmed by the occurrence of bacterial canker of hazelnut in Serbia (Popović Milovanović et al. 2026).

Early autumn frost also promotes symptom development in strains associated with bacterial shoot blight of tea (Tomihama et al. 2009). Interestingly, in this pathosystem, a clear association was observed between ice nucleation‐active strains of *Xanthomonas campestris* on the leaf surface (particularly around stomata) and pseudomonads. These *X. campestris* strains, by causing damage to plant tissues, facilitate colonisation and systemic invasion by pseudomonads already present on the phylloplane (Tomihama et al. 2009). The disease is favoured by air temperatures ranging from 10°C to 15°C, and similarly to other pathosystems, high summer temperatures are not conducive to disease progression (Tomihama et al. 2009).

For the lineage associated with cherry and plum, tree wounds caused by frost or mechanical damage present the primary entry points, whereas leaf scars are of secondary importance (Hulin, Mansfield, et al. 2018). Strains of this lineage are also capable of systemic movement from the inoculation site and can induce gummosis from cankers (Hulin, Mansfield, et al. 2018). It is noteworthy that *C. avellana* , *Actinidia* spp. and *Prunus* spp. are host plants for taxonomically distinct phytopathogenic pseudomonads belonging to different species complexes, namely *P. avellanae* , *P. amygdali* and *P. syringae* , representing cases of convergent evolution and adaptation to the same host. European hazelnut and kiwifruit can be infected by strains of *P. avellanae* and *P. syringae* , whereas cherry and plum can be infected by strains of all three species (Gomila et al. 2017).

## 6 Detection

Pseudomonads causing cankers, as well as twig or leaf blight and dieback, can be identified using selective media, pathogenicity tests, and genetic methods (Schaad et al. 2001). Despite the growing amount of genomic information available for these pathogens, standardised diagnostic procedures remain limited. Currently, within the *P. avellanae* species complex, an official diagnostic protocol has been established only for *P. syringae* pv. *actinidiae*, with guidelines for detection and identification in different types of plant material (EPPO 2021).

All lineages within the *P. avellanae* complex can be detected in both symptomatic and asymptomatic plant tissues. Isolation is recommended as an initial step for identification and can be performed using tissues such as twigs, buds, and cankers. Bacteria can be cultured on general or semiselective media, including nutrient agar (NA), NA supplemented with 5% sucrose (NSA), NSA supplemented with antibiotics, King's B medium (KB), modified KB, as well as peptone sucrose agar (PSA) (Scortichini 1994). Colonies are levan‐positive and typically appear smooth, circular, and convex with entire margins. After 3 days of incubation on NSA, they develop as pearly‐white, mucoid, dome‐shaped colonies. On modified KB medium, colonies are generally smooth and flat, with entire to slightly lobed margins, and show a pearly‐white to yellowish coloration. In *P. syringae* pv. *morsprunorum* race 2, colony morphology may initially differ from typical strains, forming levan‐type “fried‐egg” colonies on NSA after 40–84 h. However, after 3–4 days, they develop the typical morphology of standard strains (Freigoun and Crosse 1975). Although isolation is recommended, it shows lower sensitivity than molecular methods (Janse et al. 1996; Scortichini 2012; EPPO 2021; Loreti et al. 2018).

Different phenotypic traits and molecular diagnostic tools have been proposed to characterise and differentiate *P. avellanae* populations (Janse et al. 1996; Scortichini et al. 1998, 2002, 1979, 2005, 2013; Loreti et al. 2009; Popović Milovanović et al. 2026). Strains of *P. avellanae* , *P. syringae* pv. *theae*, *P. syringae* pv. *actinidiae*, and *P. syringae* pv. *morsprunorum* race 2 share several key phenotypic characteristics, including levan production, oxidase negativity, absence of potato soft rot and arginine dehydrolase activity, together with the induction of a hypersensitive response (HR) in tobacco, which places them within LOPAT group Ia (Scortichini et al. 2002; Kałużna et al. 2016). Differentiation among *P. syringae* pathogens originating from stone fruits can be performed using GATTa test (Freigoun and Crosse 1975; Latorre and Jones 1979). However, variable results have been reported for *P. syringae* pv. *morsprunorum* race 2 (Gilbert et al. 2009). Scortichini (1994) reported that nutritional and protein profile analyses revealed slight differences between Japanese and Italian strains of *P. syringae* pv. *actinidiae*. Reliable protocols for pathogenicity testing of pseudomonads of woody origin are available. Artificial inoculation of 1‐year‐old hazelnut twigs at leaf scars in early autumn with *P. avellanae* strains reliably induces twig dieback in the following spring (Scortichini and Lazzari 1996; Scortichini 2012). The pathogenicity of *P. syringae* pv. *actinidiae* can be assessed on plantlets of *A. chinensis* (cultivars Erica, Hort16A) and *A. deliciosa* (cultivars Hayward, Soreli) (EPPO 2021), as well as by in vitro leaf‐disc assays (Prencipe et al. 2018; Brunetti et al. 2020). In plantlet assays, typical symptoms appear 6–10 days after inoculation and are monitored for up to 3 weeks, whereas in leaf‐disc assays, necrotic spots develop after 6 days and spread over 14–20 days to cover the entire disc. According to early studies (Freigoun and Crosse 1975), *P. syringae* pv. *morsprunorum* variant isolates infected leaf scars of 4‐year‐old cv. Roundel trees, mostly producing localised lesions, whereas typical strains caused progressive branch lesions and bud death. For inoculation purposes, wound inoculation is recommended, as variant isolates were generally more virulent. They produced longer cankers and more severe branch dieback with specificity for cv. Roundel (Freigoun and Crosse 1975). Kałużna (2019) described a method based on infiltration of bacterial suspensions into leaves using a hypodermic syringe when testing pathogenic strains isolated from Cornelian cherry, which are among the closest relatives of hazelnut‐associated *P. avellanae* strains.

Serological methods, including ELISA, slide agglutination, and immunofluorescence (IF), are still widely used in plant pathogen diagnostics and have been applied to various species of genus *Pseudomonas*. However, their usefulness for the *P. avellanae* and *P. syringae* complexes from stone fruits is limited due to cross‐reactions with nonpathogenic bacteria and poor discrimination among strains (Fiori et al. 2003; Vicente et al. 2004). A rapid serological test (immunochromatographic strips), based on the use of a polyclonal antiserum against the effector HopZ5, was recently developed for *P. syringae* pv. *actinidiae* detection (Chen et al. 2018), but no interlaboratory validation data are currently available.

The majority of methods used for detecting lineages of the *P. avellanae* species complex rely on molecular tools, particularly PCR, due to their higher sensitivity and specificity (Table 2). Two populations of *P. avellanae* both isolated from hazelnut can be differentiated using BOX‐PCR and ERIC‐PCR (Scortichini 2012; Popović Milovanović et al. 2026). A specific primer set (PAV 1/PAV 22; Table 2) for conventional PCR detection of *P. avellanae* was developed by Scortichini and Marchesi (2001), based on regions of the 16S rRNA gene of strain PD 2390 that exhibited variability compared with the corresponding regions in the related species *P. syringae* pv. *syringae*. Soon after, Loreti and Gallelli (2002) developed a primer set (WA/WC; Table 2) for the detection of virulent *P. avellanae* strains, selected by comparing nucleotide sequences of the *hrpW* gene in *P. avellanae* , *P. syringae* pv. *tomato* and *P. syringae* pv. *syringae*. A TaqMan real‐time PCR assay was developed by Gervasi and Scortichini (2009) using two primers and a probe designed based on regions of the 16S rRNA gene of *P. avellanae* strain ISF 2059: the forward primer Pavel‐F, the reverse primer Pavel‐R, and the probe Pavel‐MGB (Table 2). Turco et al. (2023) developed a specific BRYT Green dye‐based qPCR assay, a sensitive tool for the detection and quantification of *P. avellanae* in field samples, based on a pangenomic analysis of the bacterial genome (Table 2).

Several molecular techniques have been developed for the detection and identification of *P. syringae* pv. *actinidiae*, including LAMP, PCR‐restriction fragment length polymorphism (PCR‐RFLP), multiplex, real‐time quantitative, and nested PCR (Rees‐George et al. 2010; Balestra et al. 2013; Biondi et al. 2013; Gallelli et al. 2014; Ruinelli et al. 2017). Rees‐George et al. (2010) developed two sets of PCR primers for conventional PCR, PsaF1/PsaR2 and PsaF3/PsaR4, designed to target a portion of the 16S–23S rDNA intertranscribed spacer (ITS) region (Table 2). Balestra et al. (2013) designed a primer pair, *P. syringae* pv. *actinidiae* F/ *P. syringae* pv. *actinidiae* R (Table 2), for a multiplex PCR assay for the detection of *P. syringae* pv. *actinidiae*. As some of these methods may yield false‐positive results, several alternative assays have been developed and validated. These include conventional duplex PCR (Gallelli et al. 2011), followed by conventional simplex and real‐time PCR (Gallelli et al. 2014), LAMP (Ruinelli et al. 2017), and real‐time PCR (Andersen et al. 2018). These methods were validated by EPPO (2021) and confirmed to detect *P. syringae* pv. *actinidiae* biovars 1, 2, and 3 (Table 2). Recently, Luo et al. (2025) developed a propidium monoazide (PMA)‐PCR assay for the detection of viable cells of *P. syringae* pv. *actinidiae*. Strain‐specific primers targeting the *hopZ3* effector gene were combined with the nucleic acid‐binding dye PMA to exclude DNA from dead cells. The method showed high sensitivity (detection limit: 10 CFU/mL or 1 pg/μL) and was successfully applied to infected kiwifruit tissues, providing a rapid and reliable alternative to conventional PCR for detecting viable *P. syringae* pv. *actinidiae*. Kałużna et al. (2016) developed primer sets for the detection of *P. syringae* pv. *morsprunorum* race 2: Psm2‐8F/Psm2‐8R for conventional PCR and Psm2‐1F‐RT/Psm2‐1R‐RT for real‐time PCR (Table 2).

Phylogenetic analysis of lineages within the *P. avellanae* species complex can be performed using MLSA based on four housekeeping genes, following the MLSA scheme recommended for *P. syringae: gapA* (glyceraldehyde‐3‐phosphate dehydrogenase), *gyrB* (DNA gyrase subunit B), *gltA* (citrate synthase), and *rpoD* (RNA polymerase sigma factor RpoD) (Sarkar and Guttman 2004; Hwang et al. 2005; Ferrante and Scortichini 2010, 2011; Kałużna et al. 2010). More recently, Iličić et al. (2022) recommended the use of *recG* (ATP‐dependent DNA helicase RecG) for the identification and characterisation of the *P. syringae* species complex. Subsequently, Popović Milovanović et al. (2026) demonstrated that this gene can also be used in phylogenetic analyses of hazelnut‐associated *P. avellanae* . Assignment of biovars of *P. syringae* pv. *actinidiae* is possible using a scheme based on seven genes (*acnB, cts, gapA, gyrB, pfk, pgi*, and *rpoD*) (Sarkar and Guttman 2004; Sawada et al. 2014).

For many years, identification of canker‐causing pseudomonads has largely relied on the detection of genes involved in the production of specific virulence factors, including the toxins coronatine and syringomycin, as well as the siderophore yersiniabactin. Within the *P. avellanae* species complex, the siderophore yersiniabactin (*irp1* gene) has been detected in *P. avellanae* (Marcelletti and Scortichini 2015) and *P. syringae* pv. *morsprunorum* race 2 (Bultreys et al. 2006). The *cfl* gene responsible for coronatine synthesis has been reported in *P. syringae* pv. *actinidiae*. In addition, *P. syringae* pv. *actinidiae* possesses the *tox–argK* gene encoding phaseolotoxin (Templeton et al. 2005). Within four genetically distinct populations of *P. syringae* pv. *actinidiae*, Psa1 and Psa2 produce phaseolotoxin and coronatine, respectively, whereas Psa3 and Psa4 do not produce these toxins (Scortichini 2012; Vanneste et al. 2013; Abelleira et al. 2014).

Scortichini et al. (2002) found that ARDRA, repetitive PCR using ERIC, BOX and REP primer sets, whole‐cell protein analysis, biochemical and nutritional tests, and pathogenicity tests can be used for the identification and comparison of *P. avellanae* , *P. syringae* pv. *theae* and *P. syringae* pv. *actinidiae*. The rapid progress of high‐throughput sequencing technologies, particularly next‐generation and third‐generation sequencing (NGS and TGS), has greatly expanded the possibilities for comparative genomic studies. Earlier research relied mainly on molecular markers derived from a limited number of conserved housekeeping genes or protein profiles, providing only partial insight into genetic diversity. In contrast, the increasing availability of complete genome sequences now enables genome‐wide comparisons among strains. Such genome‐scale analyses, including pangenome studies, have significantly improved our understanding of the genetic basis of pathogenicity and host adaptation, revealing factors that may facilitate specialisation on the same host or, in some cases, enable pathogens to colonise novel host plants (Marcelletti and Scortichini 2015, 2019).

## 7 Disease Control

In general, sites characterised by frequent winter and spring frost should be avoided for the cultivation of host plants susceptible to the *P. avellanae* species complex. In addition, certain agronomic practices may strongly favour disease progression, such as excessive nitrogen fertilisation, iron deficiency, and water stress (Mauri et al. 2016). Disease management should primarily focus on protecting the main points of entry into the plant (i.e., wounds, leaf scars, stomata) during periods conducive to disease development (i.e., late winter, spring, and autumn), as well as on the use of healthy propagative plant material. Although several models based on climatic parameters and pathogen biology have been developed to assess the potential global suitability of geographical areas for future epidemics (Narouei‐Khandan et al. 2022), fine‐tuned disease prediction at a local scale, based on weather forecasts, is still not available for these different lineages. This presents a limitation for the effective management of diseases causing significant economic losses. It should also be noted that this species complex has an endophytic phase during a substantial part of its life cycle, which further complicates the achievement of effective crop protection. Nevertheless, several strategies, when combined, may contribute to symptom mitigation. For bacterial canker of kiwifruit, management is mainly based on the application of copper‐based compounds in early spring to reduce epiphytic colonisation of new leaves. These compounds are also widely applied after harvest, during leaf fall, and following hail events, heavy rain, and winter frost, in order to protect petioles, leaf scars, and wounds (Cameron and Sarojini 2014). Pruning cuts should be protected with copper as well.

However, due to their phytotoxicity, pathogen penetration through flowers cannot be effectively prevented. In addition, a major concern is the increasing occurrence of copper‐resistant strains, which have frequently been isolated from kiwifruit orchards subjected to repeated copper applications (Colombi et al. 2017; Cesari et al. 2023; Thomidis et al. 2025). The use of chitosan‐based compounds may present an alternative to replace or complement copper applications (Scortichini 2014). Biocontrol agents also appear promising, either for flower protection or for limiting epiphytic colonisation by the pathogen. Some strains of *Bacillus* spp., namely *B. amyloliquefaciens* D747 and *B. subtilis* QST703, are already used as commercial products. An interesting strategy involves the selection of protective strains from the native kiwifruit microflora, such as *P. azotoformans* and *P. putida* isolated from *Actinidia* spp., which produce effective siderophores (Correia et al. 2025). The advantage of this approach lies in the use of microorganisms already adapted to common field management practices, which are capable of efficient host colonisation. *Lactobacillus plantarum* is another promising biocontrol agent, capable of preventing the epiphytic colonisation through pH reduction associated with lactic acid production (Daranas et al. 2019).

Natural products, endophytic microflora, and bacteriophages also offer promising opportunities for managing bacterial canker of kiwifruit. *Adesmia balsamica* and *Leptospermum scoparium* may provide plant extracts and endophytic microflora (i.e., *Pseudomonas* spp.), respectively, capable of reducing in vivo growth of the pathogenic strains associated with bacterial canker of hazelnut (Wicaksono et al. 2018; Díaz et al. 2025). Lytic phages and phage cocktails, including dual and triple combinations, are also being extensively investigated for the development of commercial products for the management of bacterial canker of kiwifruit, with promising results reported for in vivo symptom mitigation (Yu et al. 2016; Flores et al. 2020; Fiorillo et al. 2023).

Similarly to bacterial canker of kiwifruit, copper‐based compounds are widely used for the management of bacterial canker of hazelnut and cherry/plum. Their application occurs mainly in autumn to protect leaf scars (hazelnut) and in early spring to protect frost wounds. In addition, for bacterial canker of cherry, bacteriophages are currently being investigated as alternative management strategies (Rabiey et al. 2020). The search for resistant plant germplasm within wild species presents another strategy to replace highly susceptible cherry cultivars (Hulin, Vadillo‐Díeguez, et al. 2020).

## 8 Concluding Remarks and Perspective

In‐depth taxonomic studies have revealed previously unrecognised relationships among different phytopathogenic pseudomonads belonging to the *P. syringae* complex. Within this group, the *P. avellanae* species complex comprises taxonomically related yet distinct lineages capable of infecting different, botanically unrelated host plants. In addition, its occurrence is not limited to cultivated plant species, as a strain of this species complex has also been isolated from a natural habitat, namely river epilithon. Strains associated with the lineage causing bacterial canker of kiwifruit have also been detected in natural habitats linked to wild *Actinidia* spp. (McCann et al. 2017). Strains of this species complex can readily colonise plant organs and thus spread to different cultivation areas through the movement of propagative plant material, as observed for the pandemic strain of bacterial canker of kiwifruit (Butler et al. 2013; McCann et al. 2017), as well as for outbreaks of bacterial canker of European hazelnut in Italy and Serbia (Scortichini and Tropiano 1994; Popović Milovanović et al. 2026).

The global circulation of plant material presents a major pathway for the worldwide spread of these diseases. Effective and specific detection techniques are therefore essential for the rapid identification of latently infected plant material at borders as well as for detecting initial infection foci in newly affected areas. The ability to colonise host plants through endophytic spread is another key feature shared by lineages within the *P. avellanae* species complex. Lineages associated with bacterial canker of European hazelnut and kiwifruit can extensively colonise the entire plant, whereas the lineage causing bacterial shoot blight of tea can systemically colonise leaves and twigs. This capability may lead to rapid host plant decline and death. The lineage associated with bacterial canker of cherry is characterised by a strong capacity to colonise branch parenchyma following penetration through wounds (Freigoun and Crosse 1975). These characteristics present a major constraint for effective field management and highlight the need to develop alternative strategies capable of replacing copper‐based compounds, particularly those targeting the pathogen within plant tissues. Modern disease management approaches, including biocontrol agents, bacteriophages, nanoparticles, and disease forecasting models, may help reduce the impact of the *P. avellanae* species complex.

## Author Contributions

**Marco Scortichini:** conceptualization, supervision, visualization, resources, writing – original draft, writing – review and editing, data curation. **Tatjana Popović Milovanović:** conceptualization, writing – original draft, writing – review and editing, supervision. **Renata Iličić:** writing – original draft, writing – review and editing, supervision.

## Funding

The authors acknowledge support from the Ministry of Education, Science and Technological Development, Republic of Serbia (contract numbers 451‐03‐33/2026‐03/200010 and 451‐03‐33/2026‐03/200117).

## Conflicts of Interest

The authors declare no conflicts of interest.

## Data Availability

Data sharing is not applicable as no new data were generated.

## References

[mpp70357-bib-0001] Abelleira, A. , A. Ares , O. Aguín , A. Picoaga , M. M. López , and P. Mansilla . 2014. “Current Situation and Characterization of *Pseudomonas syringae* pv. *actinidiae* on Kiwifruit in Galicia (Northwest Spain).” Plant Pathology 63: 691–699.

[mpp70357-bib-0002] Andersen, M. T. , M. D. Templeton , J. Rees‐George , et al. 2018. “Highly Specific Assays to Detect Isolates of *Pseudomonas syringae* pv. *actinidiae* Biovar 3 and *Pseudomonas syringae* pv. *actinidifoliorum* Directly From Plant Material.” Plant Pathology 67, no. 5: 1220–1230.

[mpp70357-bib-0003] Balestra, G. M. , M. C. Taratufolo , B. A. Vinatzer , and A. Mazzaglia . 2013. “A Multiplex PCR Assay for Detection of *Pseudomonas syringae* pv. *actinidiae* and Differentiation of Populations With Different Geographic Origin.” Plant Disease 97: 472–478. 10.1094/PDIS-06-12-0590-RE 30722225

[mpp70357-bib-0004] Berge, O. , C. L. Monteil , C. Bartoli , et al. 2014. “A User's Guide to a Database of the Diversity of *Pseudomonas syringae* and Its Application to Classifying Strains in This Phylogenetic Complex.” PLoS One 9: e105547. 10.1371/journal.pone.0105547 PMC4153583 25184292

[mpp70357-bib-0005] Biondi, E. , A. Galeone , N. Kuzmanovic , S. Ardizzi , C. Lucchese , and A. Bertaccini . 2013. “ *Pseudomonas syringae* pv. *actinidiae* Detection in Kiwifruit Plant Tissue and Bleeding Sap.” Annals of Applied Biology 162: 60–70.

[mpp70357-bib-0006] Brunetti, A. , N. Pucci , V. Modesti , et al. 2020. “In Vitro and In Planta Screening of Compounds for the Control of *Pseudomonas syringae* pv. *actinidiae* in *Actinidia chinensis* var. *chinensis* .” European Journal of Plant Pathology 158: 829–848.

[mpp70357-bib-0007] Bultreys, A. , I. Gheysen , and E. de Hoffmann . 2006. “Yersiniabactin Production by *Pseudomonas syringae* and *Escherichia coli*, and Description of a Second Yersiniabactin Locus Evolutionary Group.” Applied and Environmental Microbiology 72, no. 6: 3814–3825. 10.1128/AEM.00119-06 PMC1489633 16751485

[mpp70357-bib-0008] Bultreys, A. , and M. Kaluzna . 2010. “Bacterial Canker Caused by *Pseudomonas syringae* on Fruit Species With Special Emphasis on the Pathovars *syringae* and *morsprunorum* Race 1 and Race 2.” Journal of Plant Pathology 92, no. S1: 21.

[mpp70357-bib-0009] Butler, M. I. , P. A. Stockwell , M. A. Black , R. C. Day , I. L. Lamont , and R. T. M. Poulter . 2013. “ *Pseudomonas syringae* pv. *actinidiae* From Recent Outbreaks of Kiwifruit Bacterial Canker Belong to Different Clones That Originated in China.” PLoS One 8: e57464. 10.1371/journal.pone.0057464 PMC3583860 23555547

[mpp70357-bib-0010] Cameron, A. , and V. Sarojini . 2014. “ *Pseudomonas syringae* pv. *actinidiae*: Chemical Control, Resistance Mechanisms and Possible Alternatives.” Plant Pathology 63: 1–11.

[mpp70357-bib-0011] Cesari, E. , F. Marocchi , A. L'Aurora , et al. 2023. “Occurrence of Copper‐Resistant *Pseudomonas syringae* pv. *actinidiae* Strains in Kiwifruit Orchards of Central Italy.” Journal of Phytopathology 171: 768–774.

[mpp70357-bib-0012] Chen, H. , Y. Hu , K. Qin , et al. 2018. “A Serological Approach for the Identification of the Effector HopZ5 of *Pseudomonas syringae* pv. *actinidiae*: A Tool for the Rapid Immunodetection of Kiwifruit Bacterial Canker.” Journal of Plant Pathology 100, no. 2: 171–177.

[mpp70357-bib-0013] Choi, S. , J. Jayaraman , C. Segonzac , et al. 2017. “ *Pseudomonas syringae* pv. *actinidiae* Type III Effectors Localized at Multiple Cellular Compartments Activate or Suppress Innate Immune Responses in *Nicotiana benthamiana* .” Frontiers in Plant Science 8: 2157. 10.3389/fpls.2017.02157 PMC5742410 29326748

[mpp70357-bib-0014] Colombi, E. , C. Straub , S. Künzel , M. D. Templeton , H. C. McCann , and P. B. Rainey . 2017. “Evolution of Copper Resistance in the Kiwifruit Pathogen *Pseudomonas syringae* pv. *actinidiae* Through Acquisition of Integrative Conjugative Elements and Plasmids.” Environmental Microbiology 19: 819–832. 10.1111/1462-2920.13662 28063194

[mpp70357-bib-0015] Correia, C. , A. Cellini , I. Donati , et al. 2025. “ *Pseudomonas azotoformans* and *Pseudomonas putida* : Novel Kiwifruit‐Native Biological Control Agents Against *Pseudomonas syringae* pv. *actinidiae* .” Biological Control 201: 105706.

[mpp70357-bib-0016] Daranas, N. , G. Roselló , J. Cabrefiga , et al. 2019. “Biological Control of Bacterial Plant Diseases With *Lactobacillus plantarum* Strains Selected for Their Broad‐Spectrum Activity.” Annals of Applied Biology 174: 92–105. 10.1111/aab.12476 PMC6334523 30686827

[mpp70357-bib-0017] Díaz, K. , D. Giménez , C. González , et al. 2025. “Control of Bacterial Canker Caused by *Pseudomonas syringae* pv. *actinidiae* in Kiwifruit Plants by Resinous Exudates and Extracts of *Adesmia balsamica* .” Pest Management Science 81: 5484–5498. 10.1002/ps.8907 40391566

[mpp70357-bib-0018] Donati, I. , A. Cellini , D. Sangiorgio , et al. 2020. “ *Pseudomonas syringae* pv. *actinidiae*: Ecology, Infection Dynamics and Disease Epidemiology.” Microbial Ecology 80: 81–102. 10.1007/s00248-019-01459-8 PMC7223186 31897570

[mpp70357-bib-0019] Doudoroff, M. , and N. Palleroni . 1974. “Gram‐Negative Aerobic Rods and Cocci. Genus I. *Pseudomonas* .” In Bergey's Manual of Determinative Bacteriology, 217–243. Williams and Wilkins.

[mpp70357-bib-0020] EPPO . 2021. “PM 7/120 (2) *Pseudomonas syringae* pv. *actinidiae* .” EPPO Bulletin 51: 549–567.

[mpp70357-bib-0021] Ferrante, P. , and M. Scortichini . 2010. “Molecular and Phenotypic Features of *Pseudomonas syringae* pv. *actinidiae* Isolated During Recent Epidemics of Bacterial Canker on Yellow Kiwifruit ( *Actinidia chinensis* ) in Central Italy.” Plant Pathology 59: 954–962.

[mpp70357-bib-0022] Ferrante, P. , and M. Scortichini . 2011. “Molecular and Phenotypic Variability Among *Pseudomonas avellanae* , *P. syringae* pv. *actinidiae* and *P. syringae* pv. *theae*: The Genomospecies 8 Sensu Gardan Et al. (1999).” Journal of Plant Pathology 93, no. 3: 659–666.

[mpp70357-bib-0023] Ferrante, P. , and M. Scortichini . 2014. “Frost Promotes the Pathogenicity of *Pseudomonas syringae* pv. *actinidiae* in *Actinidia deliciosa* and *A. chinensis* Plants.” Plant Pathology 63: 12–19.

[mpp70357-bib-0024] Fiori, M. , L. Cicconi , and M. Scortichini . 2003. “Bacterial Canker of Hazelnut ( *Corylus avellana* L.) in Sardinia (Italy): Occurrence of *Pseudomonas syringae* Strains.” In Pseudomonas syringae and Related Pathogens, edited by N. S. Iacobellis , A. Collmer , S. W. Hutcheson , et al., 617–625. Springer.

[mpp70357-bib-0025] Fiorillo, A. , D. Frezza , G. di Lallo , and S. Visconti . 2023. “A Phage Therapy Model for the Prevention of *Pseudomonas syringae* pv. *actinidiae* Infection of Kiwifruit Plants.” Plant Disease 107: 267–271. 10.1094/PDIS-02-22-0348-SC 35939740

[mpp70357-bib-0026] Flores, O. , J. Retamales , M. Núñez , et al. 2020. “Characterization of Bacteriophages Against *Pseudomonas syringae* pv. *actinidiae* With Potential Use as Natural Antimicrobials in Kiwifruit Plants.” Microorganisms 8: 974. 10.3390/microorganisms8070974 PMC7409275 32610695

[mpp70357-bib-0027] Freigoun, S. O. , and J. E. Crosse . 1975. “Host Relations and Distribution of a Physiological and Pathological Variant of *Pseudomonas morsprunorum* .” Annals of Applied Biology 81: 317–330.

[mpp70357-bib-0028] Fujikawa, T. , and H. Sawada . 2019. “Genome Analysis of *Pseudomonas syringae* pv. *actinidiae* Biovar 6, Which Produces the Phytotoxins Phaseolotoxin and Coronatine.” Scientific Reports 9: 3836. 10.1038/s41598-019-40754-9 PMC6405952 30846809

[mpp70357-bib-0029] Gallelli, A. , A. L'Aurora , and S. Loreti . 2011. “Gene Sequence Analysis for the Molecular Detection of *Pseudomonas syringae* pv. *actinidiae*: Developing Diagnostic Protocols.” Journal of Plant Pathology 93: 425–435.

[mpp70357-bib-0030] Gallelli, A. , S. Talocci , M. Pilotti , and S. Loreti . 2014. “Real‐Time and Qualitative PCR for Detecting *Pseudomonas syringae* pv. *actinidiae* Isolates Causing Recent Outbreaks of Kiwifruit Bacterial Canker.” Plant Pathology 63: 264–276.

[mpp70357-bib-0031] Gardan, L. , H. Shafik , S. Belouin , R. Brosch , F. Grimont , and P. A. D. Grimont . 1999. “DNA Relatedness Among the Pathovars of *Pseudomonas syringae* and Description of *Pseudomonas tremae* sp. nov. and *Pseudomonas cannabina* sp. nov.” International Journal of Systematic Bacteriology 49: 469–478. 10.1099/00207713-49-2-469 10319466

[mpp70357-bib-0032] Gervasi, F. , and M. Scortichini . 2009. “Detection of *Pseudomonas avellanae* From Hazelnut Twigs by TaqMan Real‐Time PCR.” Journal of Plant Pathology 91: 573–578.

[mpp70357-bib-0033] Gilbert, V. , F. Legros , H. Maraite , and A. Bultreys . 2009. “Genetic Analyses of *Pseudomonas syringae* Isolates From Belgian Fruit Orchards Reveal Genetic Variability and Isolate–Host Relationships Within the Pathovar *syringae*, and Help Identify Both Races of the Pathovar *morsprunorum* .” European Journal of Plant Pathology 124, no. 2: 199–218.

[mpp70357-bib-0034] Gomila, M. , A. Busquets , M. Mulet , E. García‐Valdés , and J. Lalucat . 2017. “Clarification of Taxonomic Status Within the *Pseudomonas syringae* Species Group Based on a Phylogenomic Analysis.” Frontiers in Microbiology 8: 2422. 10.3389/fmicb.2017.02422 PMC5725466 29270162

[mpp70357-bib-0035] Hori, S. 1915. “An Important Disease of Tea Plants Caused by a Bacterium.” Journal of Plant Protection 2: 1–7.

[mpp70357-bib-0036] Hulin, M. T. , A. D. Armitage , J. G. Vicente , et al. 2018. “Comparative Genomics of *Pseudomonas syringae* Reveals Convergent Gene Gain and Loss Associated With Specialization Onto Cherry ( *Prunus avium* ).” New Phytologist 219: 672–696. 10.1111/nph.15182 29726587

[mpp70357-bib-0037] Hulin, M. T. , R. W. Jackson , R. J. Harrison , and J. W. Mansfield . 2020. “Cherry Picking by Pseudomonads: After a Century of Research on Canker, Genomics Provides Insights Into the Evolution of Pathogenicity Towards Stone Fruits.” Plant Pathology 69: 962–978. 10.1111/ppa.13189 PMC7386918 32742023

[mpp70357-bib-0038] Hulin, M. T. , J. W. Mansfield , P. Brain , X. Xu , R. W. Jackson , and R. J. Harrison . 2018. “Characterization of the Pathogenicity of Strains of *Pseudomonas syringae* Towards Cherry and Plum.” Plant Pathology 67: 1177–1193. 10.1111/ppa.12834 PMC5993217 29937581

[mpp70357-bib-0039] Hulin, M. T. , A. Vadillo‐Díeguez , F. Cossu , et al. 2020. “Identifying Resistance in Wild and Ornamental Cherry Towards Bacterial Canker Caused by *Pseudomonas syringae* .” Plant Pathology 71: 949–965. 10.1111/ppa.13513 PMC9305585 35909801

[mpp70357-bib-0040] Hwang, M. S. , R. L. Morgan , S. F. Sarkar , P. W. Wang , and D. S. Guttman . 2005. “Phylogenetic Characterization of Virulence and Resistance Phenotypes of *Pseudomonas syringae* .” Applied and Environmental Microbiology 71: 5182–5191. 10.1128/AEM.71.9.5182-5191.2005 PMC1214625 16151103

[mpp70357-bib-0041] Iličić, R. , A. Jelušić , S. Marković , G. Barać , F. Bagi , and T. Popović . 2022. “ *Pseudomonas cerasi*, the New Wild Cherry Pathogen in Serbia and the Potential Use of *recG* Helicase in Bacterial Identification.” Annals of Applied Biology 180: 140–150.

[mpp70357-bib-0042] Janse, J. D. , M. P. Rossi , L. Angelucci , et al. 1996. “Reclassification of *Pseudomonas syringae* pv. *avellanae* as *Pseudomonas avellanae* (sp. nov.), the Bacterium Causing Canker of Hazelnut ( *Corylus avellana* L.).” Systematic and Applied Microbiology 19: 589–595.

[mpp70357-bib-0043] Kałużna, M. 2019. “Characterization and Phylogeny of a Novel Taxon of *Pseudomonas* spp. Closely Related to *Pseudomonas avellanae* as Causal Agent of Bacterial Leaf Blight of Cornelian Cherry ( *Cornus mas* ) and *Pseudomonas syringae* pv. *syringae* as a Pathogen of Red Dogwood ( *Cornus sanguinea* ).” Journal of Plant Pathology 101: 251–261.

[mpp70357-bib-0044] Kałużna, M. , P. Albuquerque , F. Tavares , P. Sobiczewski , and J. Puławska . 2016. “Development of SCAR Markers for Rapid and Specific Detection of *Pseudomonas syringae* pv. *morsprunorum* Races 1 and 2 Using Conventional and Real‐Time PCR.” Applied Microbiology and Biotechnology 100: 3693–3711. 10.1007/s00253-016-7295-0 PMC4803819 26830104

[mpp70357-bib-0045] Kałużna, M. , P. Ferrante , P. Sobiczewski , and M. Scortichini . 2010. “Characterization and Genetic Diversity of *Pseudomonas syringae* From Stone Fruits and Hazelnut Using Repetitive‐PCR and MLST.” Journal of Plant Pathology 92: 781–787.

[mpp70357-bib-0046] Latorre, B. A. , and A. L. Jones . 1979. “ *Pseudomonas morsprunorum*, the Cause of Bacterial Canker of Sour Cherry in Michigan and Its Epiphytic Association With *Pseudomonas syringae* .” Phytopathology 69: 335–339.

[mpp70357-bib-0047] Loreti, S. , A. Cunty , N. Pucci , et al. 2018. “Performance of Diagnostic Tests for the Detection and Identification of *Pseudomonas syringae* pv. *actinidiae* From Woody Samples.” European Journal of Plant Pathology 152: 657–676.

[mpp70357-bib-0048] Loreti, S. , and A. Gallelli . 2002. “Rapid and Specific Detection of Virulent *Pseudomonas avellanae* Strains by PCR Amplification.” European Journal of Plant Pathology 108: 237–244.

[mpp70357-bib-0049] Loreti, S. , A. Gallelli , D. de Simone , and A. Bosco . 2009. “Detection of *Pseudomonas avellanae* and the Bacterial Microflora of Hazelnut Affected by ‘Moria’ in Central Italy.” Journal of Plant Pathology 91, no. 2: 365–373.

[mpp70357-bib-0050] Luo, Y. , W. Liao , Y. Li , et al. 2025. “A Rapid and Reliable Propidium Monoazide Polymerase Chain Reaction for Detecting Viable *Pseudomonas syringae* pv. *actinidiae* .” Current Issues in Molecular Biology 47: 103. 10.3390/cimb47020103 PMC11853844 39996824

[mpp70357-bib-0051] Manceau, C. , and C. Brin . 2003. “Pathovars of *Pseudomonas syringae* Are Structured in Genetic Populations Allowing the Selection of Specific Markers for Their Detection in Plant Samples.” In Pseudomonas syringae and Related Pathogens, edited by N. S. Iacobellis , A. Collmer , S. W. Hutcheson , et al., 503–512. Springer.

[mpp70357-bib-0052] Marcelletti, S. , P. Ferrante , M. Petriccione , G. Firrao , and M. Scortichini . 2011. “ *Pseudomonas syringae* pv. *actinidiae* Draft Genomes Comparison Reveals Strain‐Specific Features Involved in Adaptation and Virulence to *Actinidia* Species.” PLoS One 6: e27297. 10.1371/journal.pone.0027297 PMC3223175 22132095

[mpp70357-bib-0053] Marcelletti, S. , and M. Scortichini . 2014. “Definition of Plant Pathogenic *Pseudomonas* Genomospecies of the *Pseudomonas syringae* Complex Through Multiple Comparative Approaches.” Phytopathology 104: 1274–1282. 10.1094/PHYTO-12-13-0344-R 24875383

[mpp70357-bib-0054] Marcelletti, S. , and M. Scortichini . 2015. “Comparative Genomic Analyses of Multiple *Pseudomonas* Strains Infecting *Corylus avellana* Reveal the Occurrence of Two Genetic Clusters With Both Common and Distinctive Virulence and Fitness Traits.” PLoS One 10: e0131112. 10.1371/journal.pone.0131112 PMC4492584 26147218

[mpp70357-bib-0055] Marcelletti, S. , and M. Scortichini . 2019. “Some Strains That Have Converged to Infect *Prunus* spp. Trees Are Members of Distinct *Pseudomonas syringae* Genomospecies and Ecotypes as Revealed by In Silico Genomic Comparison.” Archives of Microbiology 201: 67–80. 10.1007/s00203-018-1573-4 30229267

[mpp70357-bib-0056] Mauri, S. , A. Cellini , G. Buriani , I. Donati , G. Costa , and F. Spinelli . 2016. “Optimization of Cultural Practices to Reduce the Development of *Pseudomonas syringae* pv. *actinidiae*, Causal Agent of the Bacterial Canker of Kiwifruit.” Journal of Berry Research 6: 355–371.

[mpp70357-bib-0057] McAtee, P. A. , L. Brian , B. Curran , et al. 2018. “Re‐Programming of *Pseudomonas syringae* pv. *actinidiae* Gene Expression During Early Stages of Infection of Kiwifruit.” BMC Genomics 19: 822. 10.1186/s12864-018-5197-5 PMC6238374 30442113

[mpp70357-bib-0058] McCann, H. C. , L. Li , Y. Liu , et al. 2017. “Origin and Evolution of the Kiwifruit Canker Pandemic.” Genome Biology and Evolution 9: 932–944. 10.1093/gbe/evx055 PMC5388287 28369338

[mpp70357-bib-0059] Morris, C. E. , L. L. Kinkel , X. Kun , P. Prior , and D. C. Sands . 2007. “A Surprising Niche for the Plant Pathogen *Pseudomonas syringae* .” Infection, Genetics and Evolution 7: 84–92. 10.1016/j.meegid.2006.05.002 16807133

[mpp70357-bib-0060] Mulet, M. , M. Gomila , C. Scotta , D. Sánchez , J. Lalucat , and E. García‐Valdés . 2012. “Concordance Between Whole‐Cell Matrix‐Assisted Laser‐Desorption/Ionization Time‐Of‐Flight Mass Spectrometry and Multilocus Sequence Analysis Approaches in Species Discrimination Within the Genus *Pseudomonas* .” Systematic and Applied Microbiology 35: 455–464. 10.1016/j.syapm.2012.08.007 23140936

[mpp70357-bib-0061] Narouei‐Khandan, H. A. , S. P. Worner , S. L. Viljanen , A. H. van Bruggen , G. M. Balestra , and E. Jones . 2022. “The Potential Global Climate Suitability of Kiwifruit Bacterial Canker Disease ( *Pseudomonas syringae* pv. *actinidiae* (Psa)) Using Three Modelling Approaches: CLIMEX, MaxEnt and Multimodel Framework.” Climate 10, no. 2: 14.

[mpp70357-bib-0062] Nowell, R. W. , B. E. Laue , P. M. Sharp , and S. Green . 2016. “Comparative Genomics Reveals Genes Significantly Associated With Woody Hosts in the Plant Pathogen *Pseudomonas syringae* .” Molecular Plant Pathology 17: 1409–1424. 10.1111/mpp.12423 PMC5132102 27145446

[mpp70357-bib-0063] Nunes da Silva, M. , M. G. Santos , M. W. Vasconcelos , and S. M. P. Carvalho . 2022. “Mitigation of Emergent Bacterial Pathogens Using *Pseudomonas syringae* pv. *actinidiae* as a Case Study—From Orchard to Gene and Everything in Between.” Crops 2: 351–377.

[mpp70357-bib-0064] Okabe, N. , and M. Goto . 1955. “Bacterial Plant Diseases in Japan. IV. Studies on Bacterial Shoot Blight of Tea Plants Caused by *Pseudomonas theae* sp. nov.” Reports of the Faculty of Agriculture, Shizuoka University 5: 96–99.

[mpp70357-bib-0065] Petriccione, M. , A. M. Salzano , I. Di Cecco , A. Scaloni , and M. Scortichini . 2014. “Proteomic Analysis of the *Actinidia deliciosa* Leaf Apoplast During Biotrophic Colonization by *Pseudomonas syringae* pv. *actinidiae* .” Journal of Proteomics 101: 43–62. 10.1016/j.jprot.2014.01.030 24530627

[mpp70357-bib-0066] Popović Milovanović, T. , S. Loreti , P. Milovanović , et al. 2026. “ *Pseudomonas avellanae* Causing European Hazelnut Decline in Serbia.” Phytopathologia Mediterranea 65: 15–24.

[mpp70357-bib-0067] Prencipe, S. , M. L. Gullino , and D. Spadaro . 2018. “ *Pseudomonas syringae* pv. *actinidiae* Isolated From *Actinidia chinensis* var. *deliciosa* in Northern Italy: Genetic Diversity and Virulence.” European Journal of Plant Pathology 150: 191–204.

[mpp70357-bib-0068] Psallidas, P. G. 1993. “ *Pseudomonas syringae* pv. *avellanae* Pathovar Nov., the Bacterium Causing Canker Disease on *Corylus avellana* .” Plant Pathology 42: 358–363.

[mpp70357-bib-0069] Psallidas, P. G. , and C. G. Panagopoulos . 1979. “A Bacterial Canker of Hazelnut in Greece.” Phytopathologische Zeitschrift 94: 103–111.

[mpp70357-bib-0070] Rabiey, M. , S. R. Roy , D. Holtappels , et al. 2020. “Phage Biocontrol to Combat *Pseudomonas syringae* Pathogens Causing Disease in Cherry.” Microbial Biotechnology 13: 1428–1445. 10.1111/1751-7915.13585 PMC7415359 32383813

[mpp70357-bib-0071] Rees‐George, J. , J. L. Vanneste , D. A. Cornish , et al. 2010. “Detection of *Pseudomonas syringae* pv. *actinidiae* Using Polymerase Chain Reaction Primers Based on the 16S–23S rDNA Intertranscribed Spacer Region and Comparison With PCR Primers Based on Other Gene Regions.” Plant Pathology 59: 453–464.

[mpp70357-bib-0072] Ruinelli, M. , J. Blom , T. H. M. Smits , and J. F. Pothier . 2019. “Comparative Genomics and Pathogenicity Potential of Members of the *Pseudomonas syringae* Species Complex on *Prunus* spp.” BMC Genomics 20: 172. 10.1186/s12864-019-5555-y PMC6402114 30836956

[mpp70357-bib-0073] Ruinelli, M. , P. H. Schneeberger , P. Ferrante , et al. 2017. “Comparative Genomics‐Informed Design of Two LAMP Assays for Detection of the Kiwifruit Pathogen *Pseudomonas syringae* pv. *actinidiae* and Discrimination of Isolates Belonging to the Pandemic Biovar 3.” Plant Pathology 66, no. 1: 140–149.

[mpp70357-bib-0074] Sarkar, S. F. , and D. S. Guttman . 2004. “Evolution of the Core Genome of *Pseudomonas syringae* , a Highly Clonal Endemic Plant Pathogen.” Applied and Environmental Microbiology 70: 1999–2012. 10.1128/AEM.70.4.1999-2012.2004 PMC383139 15066790

[mpp70357-bib-0075] Sawada, H. , T. Miyoshi , and Y. Ide . 2014. “Novel MLSA Group (Psa5) of *Pseudomonas syringae* pv. *actinidiae* Causing Bacterial Canker of Kiwifruit ( *Actinidia chinensis* ) in Japan.” Japanese Journal of Phytopathology 80: 171–184.

[mpp70357-bib-0076] Schaad, N. W. , J. B. Jones , and W. Chun . 2001. Laboratory Guide for the Identification of Plant Pathogenic Bacteria. 3rd ed. American Phytopathological Society (APS Press).

[mpp70357-bib-0077] Scortichini, M. 1994. “Occurrence of *Pseudomonas syringae* pv. *actinidiae* on Kiwifruit in Italy.” Plant Pathology 43: 1035–1038.

[mpp70357-bib-0078] Scortichini, M. 2002. “Bacterial Canker and Decline of European Hazelnut.” Plant Disease 86: 704–709. 10.1094/PDIS.2002.86.7.704 30818564

[mpp70357-bib-0079] Scortichini, M. 2012. “Detection and Identification Methods and New Tests as Developed and Used in the Framework of COST 873 for Bacteria Pathogenic to Stone Fruits and Nuts.” Journal of Plant Pathology 94, no. Suppl. 1: S1.115–S1.116.

[mpp70357-bib-0080] Scortichini, M. 2014. “Field Efficacy of Chitosan to Control *Pseudomonas syringae* pv. *actinidiae*, the Causal Agent of Kiwifruit Bacterial Canker.” European Journal of Plant Pathology 140: 887–892.

[mpp70357-bib-0081] Scortichini, M. , M. T. Dettori , U. Marchesi , M. A. Palombi , and M. P. Rossi . 1998. “Differentiation of *Pseudomonas avellanae* Strains From Greece and Italy by Rep‐PCR Genomic Fingerprinting.” Journal of Phytopathology 146: 417–420.

[mpp70357-bib-0082] Scortichini, M. , and M. Lazzari . 1996. “Systemic Migration of *Pseudomonas syringae* pv. *avellanae* in Twigs and Young Shoots of Hazelnut and Symptom Development.” Journal of Phytopathology 144: 215–219.

[mpp70357-bib-0083] Scortichini, M. , S. Marcelletti , P. Ferrante , and G. Firrao . 2013. “A Genomic Redefinition of *Pseudomonas avellanae* Species.” PLoS One 8, no. 9: e75794. 10.1371/journal.pone.0075794 PMC3783423 24086635

[mpp70357-bib-0084] Scortichini, M. , and U. Marchesi . 2001. “Sensitive and Specific Detection of *Pseudomonas avellanae* Strains Using Primers Based on 16S rRNA Gene Sequences.” Journal of Phytopathology 149: 527–532.

[mpp70357-bib-0085] Scortichini, M. , U. Marchesi , and P. di Prospero . 2002. “Genetic Relatedness Among *Pseudomonas avellanae* , *Pseudomonas syringae* pv. *theae* and *P. syringae* pv. *actinidiae*, and Their Identification.” European Journal of Plant Pathology 108: 269–278.

[mpp70357-bib-0101] Scortichini, M. , M. P. Rossi , S. Loreti , et al. 2005. “ *Pseudomonas syringae* pv. *coryli* , the Causal Agent of Bacterial Twig Dieback of *Corylus avellana* .” Phytopathology 95, no. 11: 1316–1324. 10.1094/phyto-95-1316. 18943363

[mpp70357-bib-0086] Scortichini, M. , F. Spinelli , and M. T. Templeton . 2023. “The Bacterial Canker of Kiwifruit Caused by *Pseudomonas syringae* pv. *actinidiae* .” In Kiwifruit. Botany, Production and Uses, edited by A. C. Richardson , J. N. Burdon , and A. R. Ferguson , 317–331. CABI International.

[mpp70357-bib-0087] Scortichini, M. , and F. G. Tropiano . 1994. “Severe Outbreak of *Pseudomonas syringae* pv. *avellanae* on Hazelnut in Italy.” Journal of Phytopathology 140: 65–70.

[mpp70357-bib-0088] Spotts, R. A. , J. L. Olsen , L. E. Long , and J. W. Pscheidt . 2010. Bacterial Canker of Sweet Cherry in Oregon: Disease Symptoms, Cycle, and Management. Oregon State University, Extension Service. http://efaidnbmnnnibpcajpcglclefindmkaj/.

[mpp70357-bib-0089] Stolp, H. , M. P. Starr , and N. L. Baigent . 1965. “Problems in Speciation of Phytopathogenic Pseudomonads and Xanthomonads.” Annual Review of Phytopathology 3, no. 1: 231–264.

[mpp70357-bib-0090] Takikawa, Y. , S. Serizawa , T. Ichikawa , S. Tsuyumu , and M. Goto . 1989. “ *Pseudomonas syringae* pv. *actinidiae* pv. nov. the Causal Bacterium of Canker of Kiwifruit in Japan.” Japanese Journal of Phytopathology 55, no. 4: 437–444.

[mpp70357-bib-0091] Templeton, M. D. , L. A. Reinhardt , C. A. Collyer , R. E. Mitchell , and W. W. Cleland . 2005. “Kinetic Analysis of the L‐Ornithine Transcarbamoylase From *Pseudomonas savastanoi* pv. *phaseolicola* That Is Resistant to the Transition State Analogue (*R*)‐*N*Δ‐(*N*′‐Sulfodiaminophosphinyl)‐L‐Ornithine.” Biochemistry 44: 4408–4415. 10.1021/bi047432x 15766270

[mpp70357-bib-0092] Thomidis, T. , M. G. Pagoulatou , E. Alexandridis , E. Malantinaki , and D. E. Goumas . 2025. “Copper Resistance in *Pseudomonas syringae* pv. *actinidiae* Strains From Northern Greece and Evaluation of Several Commercial Products for Disease Control.” Agriculture 15: 157.

[mpp70357-bib-0093] Tomihama, T. , T. Nonaka , Y. Nishi , and K. Arai . 2009. “Environmental Control in Tea Fields to Reduce Infection by *Pseudomonas syringae* pv. *theae* .” Phytopathology 99, no. 2: 209–216. 10.1094/PHYTO-99-2-0209 19159313

[mpp70357-bib-0094] Turco, S. , M. I. Drais , R. de Vivo , V. Cristofori , and A. Mazzaglia . 2023. “Development of a Quantitative qPCR Assay for the Detection of *Pseudomonas avellanae* in Hazelnut Tissues.” Acta Horticulturae 1379: 341–348.

[mpp70357-bib-0095] Turco, S. , L. Zuppante , M. I. Drais , and A. Mazzaglia . 2022. “Dressing Like a Pathogen: Comparative Analysis of Different *Pseudomonas* Genomospecies Wearing Different Features to Infect *Corylus avellana* .” Journal of Phytopathology 170, no. 7–8: 504–516.

[mpp70357-bib-0096] Vanneste, J. L. , J. Yu , D. A. Cornish , et al. 2013. “Identification, Virulence, and Distribution of Two Biovars of *Pseudomonas syringae* pv. *actinidiae* in New Zealand.” Plant Disease 97: 708–719. 10.1094/PDIS-07-12-0700-RE 30722585

[mpp70357-bib-0097] Vicente, J. G. , J. P. Alves , K. Russell , and S. J. Roberts . 2004. “Identification and Discrimination of *Pseudomonas syringae* Isolates From Wild Cherry in England.” European Journal of Plant Pathology 110: 337–351.

[mpp70357-bib-0098] Wicaksono, W. A. , E. E. Jones , S. Casonato , J. Monk , and H. J. Ridgway . 2018. “Biological Control of *Pseudomonas syringae* pv. *actinidiae*, the Causal Agent of Bacterial Canker of Kiwifruit, Using Endophytic Bacteria Recovered From a Medicinal Plant.” Biological Control 116: 103–112.

[mpp70357-bib-0099] Young, J. M. , D. W. Dye , J. F. Bradbury , C. G. Panagopoulos , and C. F. Robbs . 1978. “A Proposed Nomenclature and Classification for Plant Pathogenic Bacteria.” New Zealand Journal of Agricultural Research 21, no. 1: 153–177.

[mpp70357-bib-0100] Yu, J.‐G. , J.‐A. Lim , Y.‐R. Song , et al. 2016. “Isolation and Characterization of Bacteriophages Against *Pseudomonas syringae* pv. *actinidiae* Causing Bacterial Canker Disease in Kiwifruit.” Journal of Microbiology and Biotechnology 26: 385–393. 10.4014/jmb.1509.09012 26628254

